# Left atrial dysfunction may precede left atrial enlargement and abnormal left ventricular longitudinal function: a cardiac MR feature tracking study

**DOI:** 10.1186/s12872-022-02532-w

**Published:** 2022-03-13

**Authors:** Di Zhou, Wenjing Yang, Yingxia Yang, Gang Yin, Shuang Li, Baiyan Zhuang, Jing Xu, Jian He, Weichun Wu, Yong Jiang, Xiaoxin Sun, Yining Wang, Arlene Sirajuddin, Shihua Zhao, Minjie Lu

**Affiliations:** 1 Department of Magnetic Resonance Imaging, Fuwai Hospital and National Center for Cardiovascular Diseases, Chinese Academy of Medical Sciences and Peking Union Medical College, No. 167 Beilishi Road, Beijing, 100037 China; 2 Department of Radiology, The People’s Hospital of Guangxi Zhuang Autonomous Region, Nanning, China; 3 Department of Echocardiography, Fuwai Hospital and National Center for Cardiovascular Diseases, Chinese Academy of Medical Sciences and Peking Union Medical College, Beijing, China; 4 Department of Nuclear Medicine, Fuwai Hospital and National Center for Cardiovascular Diseases, Chinese Academy of Medical Sciences and Peking Union Medical College, Beijing, China; 5 National Heart, Lung and Blood Institute (NHLBI), National Institutes of Health (NIH), Bethesda, USA; 6 Key Laboratory of Cardiovascular Imaging (Cultivation), Chinese Academy of Medical Sciences, Beijing, China

**Keywords:** Cardiomyopathy, Hypertension, Hypertrophic, Magnetic resonance imaging, Atrial function

## Abstract

**Background:**

The role of the dysfunction of left atrium in the occurrence and development of cardiovascular disease has been gradually recognized. We aim to compare the impact on left atrial (LA) function between patients with hypertrophic cardiomyopathy (HCM) and hypertension (HTN) without LA enlargement using cardiovascular magnetic resonance feature tracking (CMR-FT), and if possible, explore the capability of LA function for providing clinical implication and predicting clinical adverse events in the early stage of cardiovascular disease.

**Methods:**

Consecutive 60 HCM patients and 60 HTN patients with normal LA size among 1413 patients who underwent CMR were retrospectively analyzed as well as 60 controls. Left atrial and ventricular functions were quantified by volumetric and CMR-FT derived strain analysis from long and short left ventricular view cines. The primary endpoint was a composite of all-cause death, stroke, new-onset or worsening heart failure to hospitalization, and paroxysmal or persistent atrial fibrillation.

**Results:**

Compared to the controls, both HTN and HCM participants had impaired LA reservoir function (εs) and conduit function (εe) with the different stage of LA booster pump dysfunction (εa). LA strain was more sensitive than LV longitudinal strain (GLS) for evaluate primary endpoint (εs: 33.9% ± 7.5 vs. 41.2% ± 14.3, *p* = 0.02; εe: 13.6% ± 6.2 vs. 17.4% ± 10.4, *p* = 0.03; εa: 20.2% ± 6.0 vs. 23.7% ± 8.8, *p* = 0.07; GLS: -19.4% ± 6.4 vs. -20.0% ± 6.8, *p* = 0.70, respectively). After a mean follow-up of 6.8 years, 23 patients reached primary endpoint. Cox regression analyses indicated impaired LA reservoir and booster pump strain were associated with clinical outcomes in patients at the early stage of HTN and HCM (*p* < 0.05).

**Conclusions:**

CMR-FT-derived strain is a potential and robust tool in demonstrating impaired LA mechanics, quantifying LA dynamics and underlining the impacts on LA-LV coupling in patients with HTN and HCM without LA enlargement. The corresponding LA dysfunction is a promising metric to assess clinical implication and predict prognosis at the early stage, superior to GLS.

## Background

For the last decades, left atrial (LA) size has been related to increased morbidity and adverse outcomes in hypertension (HTN) and hypertrophic cardiomyopathy (HCM) patients, including atrial fibrillation (AF), heart failure (HF) and death [1, 2]. According to current guidelines, LA size is considered as one of the significant prognostic factors for sudden cardiac death in selected populations [3–5]. However, this parameter is not only insensitive, but also often inaccurate for assessing abnormality and predicting outcome, especially in the early stages of the two diseases [4, 6, 7]. In recent years, the role of the dysfunction of left atrium in the occurrence and development of cardiovascular disease has been gradually recognized [7–9]. Previous studies have demonstrated impaired LA function prior to LA enlargement assessed by LA deformation and LA volumetric indices and is tightly associated with left ventricular hypertrophy (LVH) and LV diastolic dysfunction [4, 10]. One of the probable mechanism is that it is not a simple adaptive hypertrophy, but a complex remodeling process impacted by the responses of the non-cardiomyocytic and cardiomyocytic components of the heart to dynamic mechanical and neurohumoral stimuli [11].

During hemodynamic stress or exertion, LA serves as the modulation of LV diastolic filling and cardiac performance by reservoir, conduit, and booster pump function [12]. Cardiovascular magnetic resonance (CMR) has emerged as a robust imaging technique to provide a detailed performance of HCM and HTN, including both identification of LV remodeling and impaired function [13, 14]. LA deformation was initially studied by volumetric and strain measurements using echocardiographic speckle tracking (STE) with the advantage of detailed evaluation of LA phasic function [15]. CMR feature tracking (CMR-FT) is a novel offline approach to assess myocardial deformation from steady-state free precession (SSFP) cine CMR by the tracking of tissue voxel motion [16]. In view of the thin anatomical LA walls, CMR-FT is superior to STE considering spatial resolution, view fields and reproducibility [14, 16]. It has been reported that impaired LA function independently predicts new-onset atrial fibrillation and LA function is associated with LV outflow gradient after septal ablation or septal myectomy in patients with HCM using CMR-FT [7, 17]. However, there is limited data comprehensively focusing on the association among LA function, abnormal LA-LV coupling and prognosis in HTN and HCM patients with normal LA size.

In the current study, we aim to compare the impact on LA function between patients with HCM and HTN without LA enlargement, as assessed through simultaneous LA and LV structural and functional analyses using CMR-FT, and explore the capability of LA function for providing clinical implication and predicting clinical adverse events in HTN and HCM patients with normal LA size.

## Methods

### Study population

**Fig. 1:**
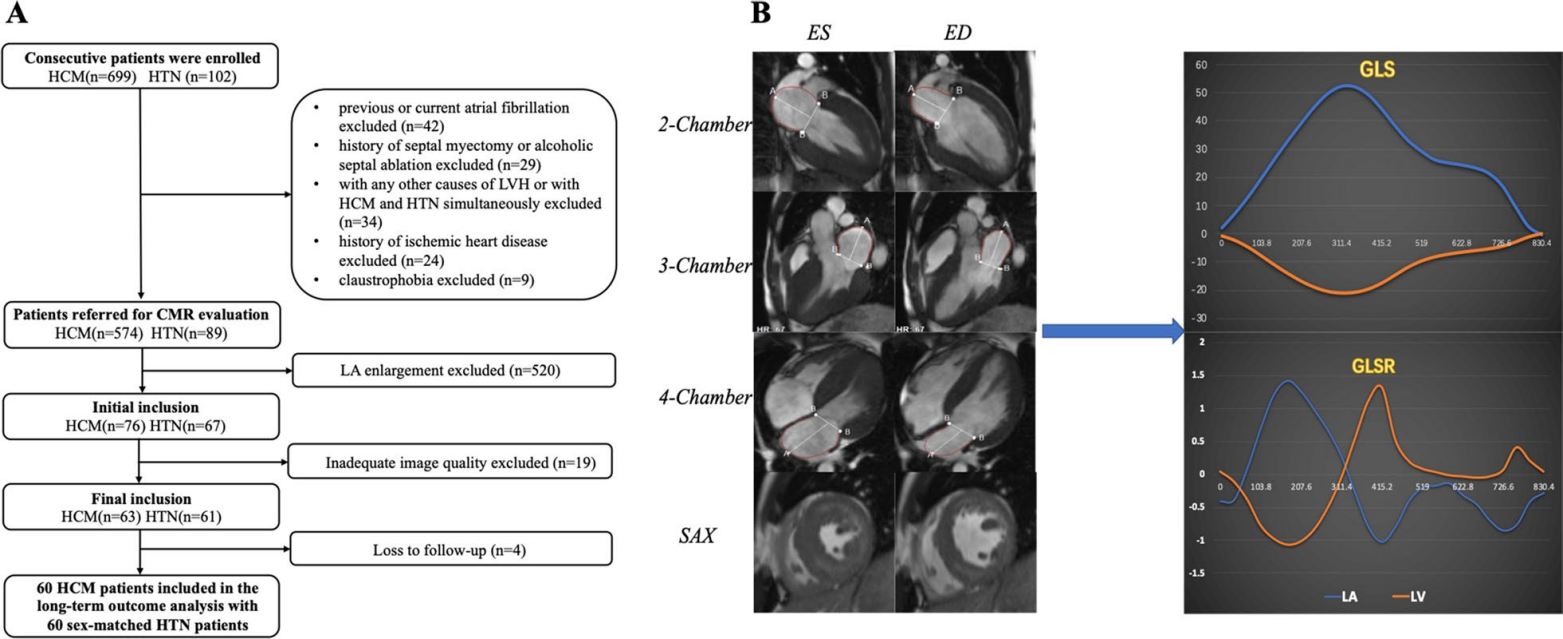
**A** Flowchart of study inclusion and exclusion. **B** This figure shows a representative example of CMR performance. HCM, hypertrophic cardiomyopathy; HTN, hypertension; CMR, Cardiac magnetic resonance; ED, end-diastole; ES, end-systole; SAX, short axis slice; LA, left atrium; LV, ventricle; GLS, global longitudinal strain; GLSR, global longitudinal strain rate

Between August 2012 and March 2016, consecutive 60 HCM patients and 60 hypertensive patients who were evaluated CMR in our hospital were retrospectively enrolled in this study with similar sex distribution (Fig. 1A). HCM was defined as maximal wall thickness ≥ 15 mm in LV myocardial segments, or ≥ 13 mm in subject with family history of HCM, without other diseases accounted for the hypertrophy [3]. Subjects were considered to be hypertensive if they had an office systolic blood pressure > 140 mmHg [5]. The exclusion criteria include the following: (a) Patients with LA enlargement which defined by the presence of LA end-diastolic volume > 95th centile of gender-specific and age-specific CMR reference ranges [18], (b) LV ejection fraction < 50%, (c) history of septal myectomy or alcoholic septal ablation, (d) history of ischemic heart disease or chronic kidney disease, (e) other conventional contraindications to CMR (e.g., previous or present of AF). A total of 60 sex-matched controls were selected and undergone complete CMR examination. None of them had evidence of metabolic or cardiovascular disease throughout the medical history in whom electrocardiograph (ECG), echocardiography, CMR and exercise testing were normal. This study was approved by the committee of our hospital and written informed consents were waived due to retrospective nature.

### CMR scan protocol

CMR images were performed at 1.5 T MR (Magnetom Avanto; Siemens, Erlangen, Germany) with 8-channel cardiac coil and retrospective ECG gating. Balanced SSFP breath-held cine images were obtained in the following planes: a 2-chamber view, a 3-chamber view, a 4-chamber view, and 8 equidistant short-axis planes covering entire LA and LV. Conventional imaging parameters included the following: slice thickness 8 mm, repetition time 2.9 ~ 3.4 ms, echo time 1.1 ~ 1.5 ms, temporal resolution: 30 ~ 55 ms, field of view 320 × 320 mm ~ 380 × 380 mm, matrix size 192 × 162 [14].

### CMR analysis

LA volume was measured by commercially available software (Qmass, Medis Suite 3.1, the Netherlands), which was obtained at late LV diastole after LA contraction (LAV_min_), at LV diastole before LA contraction (LAV_pre-a_) and at LV end-systole (LAV _max_) in 2-, 3- and 4-chamber cine views. LA emptying fraction (LAEF) were respectively calculated as follows: (a) LA total EF = (LAV_max_ − LAV_min_) × 100%/LAV_max_, (b) LA passive EF = (LAV_max_ − LAV_pre-a_) × 100%/LAV_max_, (c) LA active EF = (LAV_pre-a_ − LAV_min_) × 100%/LAV_pre-a_ [19].

Left atrial and ventricular strain and strain rate (SR) analysis were performed offline using a commercially available software (QStrain, Medis Suite 3.1, the Netherlands) based on 2-, 3- and 4-chamber cine images (Fig. 1B) [6]. LA endocardial borders were manually drawn without pulmonary veins and the LA appendage when LA was at its maximum and minimum volume. LV longitudinal strain was obtained by standard end-diastolic endo- and epicardial contours with the defining mitral valve plane and LV apex in LV 2-, 3- and 4-chamber cine balanced SSFP single-slice images. Radial and circumferential strain were obtained by standard end-diastolic endo- and epicardial contours to three selected slices at representative basal, mid-ventricular and apical levels in LV short axis cine balanced SSFP stack [20]. Then, the myocardial contours were automatically tracked throughout the entire cardiac cycle. The software feature tracking performance was visually reviewed in order to ensure accurate tracking. In cases of insufficient tracking, the software allows for border readjusted and then propagation algorithm reapplied [19]. Three aspects of LA strain were calculated from outcomes as previously mentioned: total strain (εs, first strain peak, reflective of atrial reservoir function during LV systole), active strain (εa, second strain peak, reflective of LA booster pump function during late LV diastole) and passive strain (εe, difference between εs and εa, reflective of atrial conduit function during early LV diastole), which respectively correspond to LA reservoir function, boost pump function and conduit function [14, 16, 19]. LV global longitudinal strain (GLS), as well as LA global strain, were calculated from the average of the peak stain of the corresponding three slices [16]. Consequently, three LA SR parameters were derived: total SR (SRs, peak positive strain rate), active SR (SRa, late peak negative strain rate) and passive SR (SRe, peak early negative strain rate). And then, three LV global longitudinal peak strain rate values were measured according to its curve, as following: peak GLSR during LV contraction (GLSRs), peak GLSR during early filling (GLSRd1) and peak GLSR during atrial contractility at the end of LV diastole (GLSRd2) [12].

### Follow-up

The primary endpoint was a composite of all-cause death, stroke, new-onset or worsening HF to hospitalization, and paroxysmal or persistent AF [21]. Incident HF was identified by (1) a definite diagnosis of decompensated HF; (2) pulmonary congestion proved by clinical or radiological evidence, increased LV filling pressures proved by invasive evidence, or elevated natriuretic peptide levels (NT-proBNP > 1000 ng/L or BNP [B-type natriuretic peptide] > 300 ng/L) [22]. Patients were followed with telephone interviews by two independent trained investigators who used described criteria. Medical records and copies of death certificates were requested to ascertained the incidence of clinical adverse events [22, 23].

### Reproducibility

Intra- and inter-observer variability for the LV and LA strain parameters were analyzed in a group of 30 randomly selected subjects (10 HCM subjects, 10HTN subjects and 10 controls) by two investigators who were blinded to each other’s results. The intraclass correlation coefficients (ICC) were assessed to evaluate intra- and inter-observer reproducibility. Agreement was considered excellent when ICC > 0.75, good when ICC = 0.60 ~ 0.74, fair when ICC = 0.40 ~ 0.59, and poor when ICC < 0.4 [24].

### Statistical analysis

Normally distributed continuous variables were verified using Kolmogorov–Smirnov test. Continuous variables were presented as the means ± standard deviations as appropriate. Categorical variables were expressed as numbers and percentages. Comparisons of continuous variables among three groups were performed using one-way analysis of variance (ANOVA), followed by the Tukey or Games-Howell post hoc pairwise comparison test, respectively. Categorical variables were compared using Fisher’s exact test or χ^2^ tests. Accordingly, Pearson correlation was performed to investigate the correlation between LA and LV parameters. The correlation was considered weak if r < 0.3, fair if r was between 0.3–0.5, moderate if r was between 0.5–0.7, and strong if r > 0.7 [25].

We calculated sample size based on representative metric, LA εa, using PASS (version 15), a combined standard deviation of 5% for εa measurements, and a ratio of 1:1:1 for the HCM, HTN and healthy volunteer groups [10]. Based on a one-way analysis of variance study, the result showed that sample sizes of 52, 52, and 52 are obtained from the 3 groups whose means are to be compared. The total sample of 156 subjects achieves 90% power to detect differences among the means versus the alternative of equal means using an F test with a 0.01 significance level. Therefore, we enrolled 60 participants in each group with sufficiently statistical power. For survival analyses, Univariable Cox regression models were computed to evaluate the unadjusted hazard of primary endpoint. Hazard ratios (HRs) with corresponding 95% confidence intervals were generated. Receiver operating characteristic (ROC) analyses were performed to estimate the area under the curves (AUC) and the optimal cutoff values of potential risk factors. Subsequently, the differences in event-free survival according to impaired LA strain, LV hypertrophy were compared by log-rank tests and were evaluated by Kaplan–Meier survival analysis. Parameters were stratified by the median once established cut-off values were lacking. Statistical analysis was performed by using IBM SPSS (version 22.0, Chicago). Statistical significance was defined as *p* < 0.05, which all values are 2 tailed.

## Results

### Baseline characteristics

A total of 574 HCM patients and 89 HTN patients were enrolled for screening and 520 patients with LA enlargement were excluded eventually. Another 19 patients were excluded due to the poor quality of CMR images for performing myocardial CMR-FT. Hence, 60 HCM patients and 60 HTN patients were ultimately included for analysis in this study with simple size- and sex-matched controls (Fig. 1A). There was no significant difference in gender or body surface area among the three groups. The complete baseline characteristics were presented in Table 1.

**Table 1:** Baseline clinical characteristics

	HTN (n = 60)	HCM (n = 60)	Controls (n = 60)	*p* value^‡^
*Clinical baseline*				
Age, y	49.8 ± 11.0*^†^	39.9 ± 13.5	39.6 ± 11.9	**< 0.01**
Male, n(%)	33 (55)	43 (71.7)	38 (63)	0.16
BSA, m^2^	1.8 ± 0.2	1.7 ± 0.2	1.8 ± 0.2	0.06
SBP, mmHg	157.7 ± 18.6*^†^	120.4 ± 8.3	116.2 ± 10.3	**< 0.01**
DBP, mmHg	96.3 ± 16.9*^†^	73.3 ± 8.5	73.3 ± 10.2	**< 0.01**
Family history, n(%)	18 (30)^†^	7 (11.7)		**0.01**
Diabetes, n(%)	2 (3.3)	2 (3.3)		0.81
Hyperlipidemia, n(%)	20 (33.3)	14 (23.3)		0.16
Smoker, n(%)	25 (41.7)*^†^	11 (18.3)	3 (5)	**< 0.01**
Drinking, n(%)	21 (35)*^†^	6 (10)	5 (8.3)	**< 0.01**
*Medications (%)*				
Beta blockers	27 (45)^†^	46 (76.7)		**< 0.01**
ACEI or ARB	26 (43.3)^†^	11 (18.3)		**< 0.01**
Aspirin	23 (38.3)	18 (30)		0.34
Calcium channel blockers	26 (43.3)	26 (43.3)		> 0.99
Diuretics	1 (1.7)^†^	12 (20)		**< 0.01**

Data are expressed as mean ± standard deviations or percentages in parentheses. Bold values indicate statistical significance

HTN, hypertension; HCM, hypertrophic cardiomyopathy; BSA, body surface area; SBP, systolic blood pressure; DBP, diastolic blood pressure; ACEI, angiotensin-converting enzyme inhibitor; ARB, angiotensin receptor blocker

*Indicating p < 0.05 when compared with controls;

^†^Indicating p < 0.05 when compared with HCM group;

^‡^Significance of difference among three groups

Patients with HTN was elder than that in the groups of HCM and controls. The history of smoking and drinking (both *p* < 0.01) were also higher in HTN group. There were less patients taking beta-blockers and diuretics, and more patients taking angiotensin-converting enzyme inhibitor or angiotensin receptor blocker in patients with HTN than in patients with HCM. Of the 60 HTN patients, 18 (30%) admitted with a family history of HTN while 7 (11.7%) of HCM patients involved with a family history of HCM (*p* < 0.01).

### Left ventricular structural and functional abnormality

**Fig. 2:**
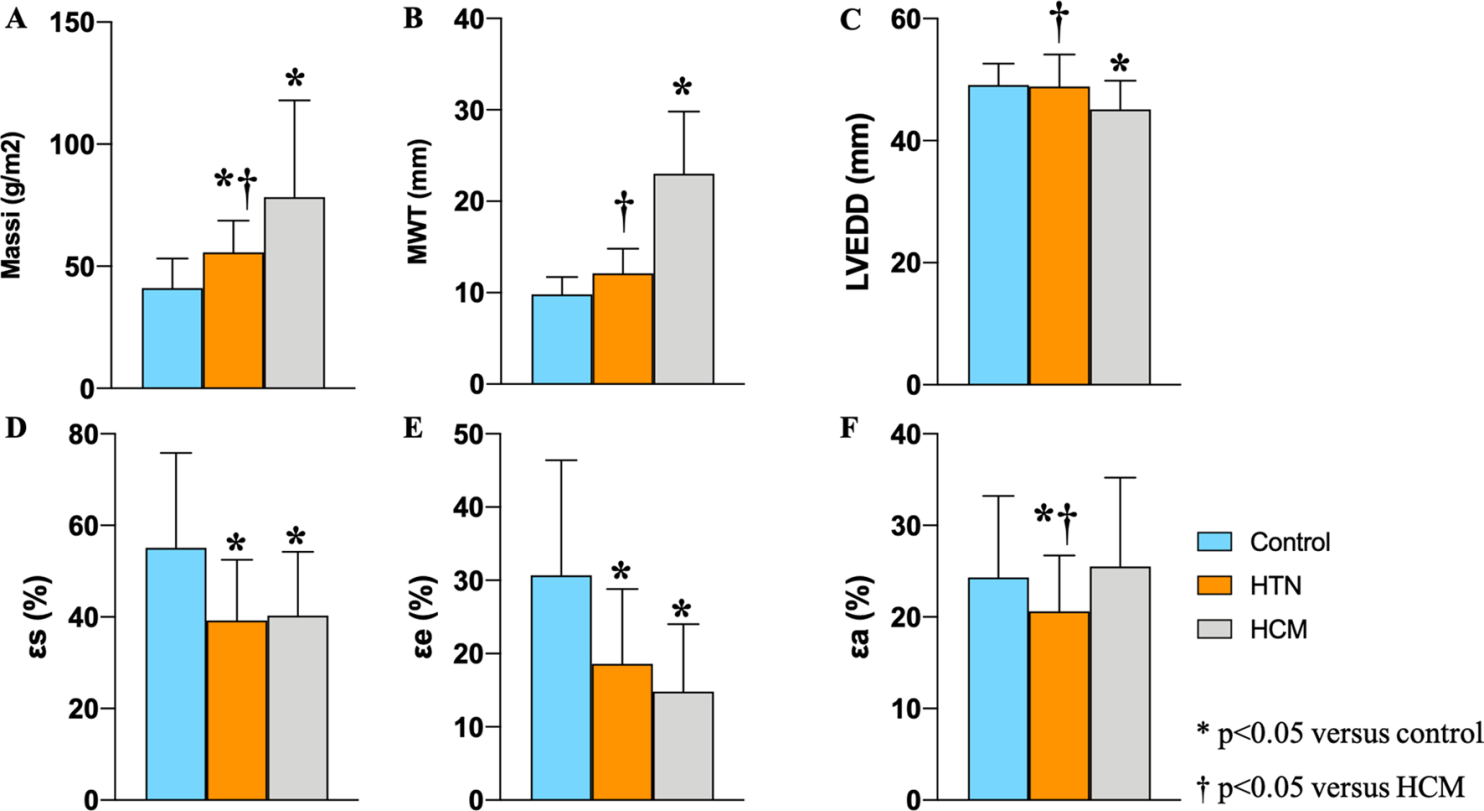
One-way ANOVA with post hoc pairwise comparison test was performed for comparisons of LV hypertrophy and LA strain among three groups. *Indicating p < 0.05 when compared with controls; †Indicating p < 0.05 when compared with HCM group; MWT, maximal wall thickness; LV, left ventricular; EDD, end-diastolic diameter; εs, total strain; εe, passive strain; εa, active strain; LA, left atrial; HCM, hypertrophic cardiomyopathy

As shown in Table 2, patients with HCM had lower LV end-diastolic diameter (controls, 49.3 mm ± 3.6; HTN patients, 48.9 mm ± 5.2; HCM patients, 45.1 mm ± 4.7, *p* < 0.01); greater LV mass index (controls, 42.5 g/m^2^ ± 12.1; HTN patients, 55.7 g/m^2^ ± 13.0; HCM patients, 78.3 g/m^2^ ± 39.6, *p* < 0.01) and LV maximum wall thickness (MWT) (controls, 9.8 mm ± 1.9; HTN patients, 12.1 mm ± 2.7; HCM patients, 23.0 mm ± 6.8, *p* < 0.01) than patients with HTN and the controls (Fig. 2). The LV mass index was also higher in HTN patients compared with the controls and no significant difference was noted in LV end-diastolic diameter and wall thickness between the HTN and control group. Impaired left ventricular GLS in patients with HCM and higher GLSRd2 in patients with HTN were observed compared to the control (all *p* < 0.01) (Table 2).

**Table 2:** LV hypertrophy, volumetric and strain parameters

	HTN (n = 60)	HCM (n = 60)	Controls (n = 60)	*p* value^‡^
*LV conventional parameters*				
EDD, mm	48.9 ± 5.2^†^	45.1 ± 4.7*	49.3 ± 3.6	**< 0.01**
EF, %	66.4 ± 10.0*^†^	62.7 ± 8.4	61.6 ± 6.0	**< 0.01**
EDVI, ml/m^2^	70.0 ± 22.6	67.0 ± 18.0	71.7 ± 20.9	0.46
ESVI, ml/m^2^	24.3 ± 8.3	25.4 ± 10.5	27.7 ± 9.7	0.14
SVI, ml/m^2^	48.0 ± 11.1^†^	41.7 ± 10.9	45.8 ± 12.0	**0.01**
CI, l/min/m^2^	3.3 ± 0.9	3.0 ± 0.8	3.1 ± 0.8	0.13
Massi, g/m^2^	55.7 ± 13.0*^†^	78.3 ± 39.6*	42.5 ± 12.1	**< 0.01**
MWT, mm	12.1 ± 2.7^†^	23.0 ± 6.8*	9.8 ± 1.9	**< 0.01**
*LV strain*				
GLS, %	− 21.1 ± 5.3	− 18.6 ± 7.7*	− 22.4 ± 4.5	**< 0.01**
GLSRs, s-1	− 1.1 ± 0.3	− 1.0 ± 0.4	− 1.0 ± 0.3	0.07
GLSRd1, s-1	0.9 ± 0.4	0.8 ± 0.4	0.9 ± 0.3	0.10
GLSRd2, s-1	0.5 ± 0.2*	0.5 ± 0.3	0.4 ± 0.2	**< 0.01**

Data are expressed as mean ± standard deviations. Bold values indicate statistical significance

HTN, hypertension; HCM, hypertrophic cardiomyopathy; LV, left ventricular; EDD, end-diastolic diameter; EF, ejection fraction; EDVI, end-diastolic volume index; ESVI, end-systolic volume index; SVI, stroke volume index; CI, cardiac index; MWT, maximal wall thickness; GLS, global longitudinal strain; GLSRs, peak global longitudinal strain rate during LV contraction; GLSRd1, peak global longitudinal strain rate during early filling; GLSRd2, peak global longitudinal strain rate during atrial contractility at the end of LV diastole

*Indicating p < 0.05 when compared with controls

^†^Indicating p < 0.05 when compared with HCM group

^‡^Significance of difference among three groups

### Left atrial dysfunction

**Fig. 3:**
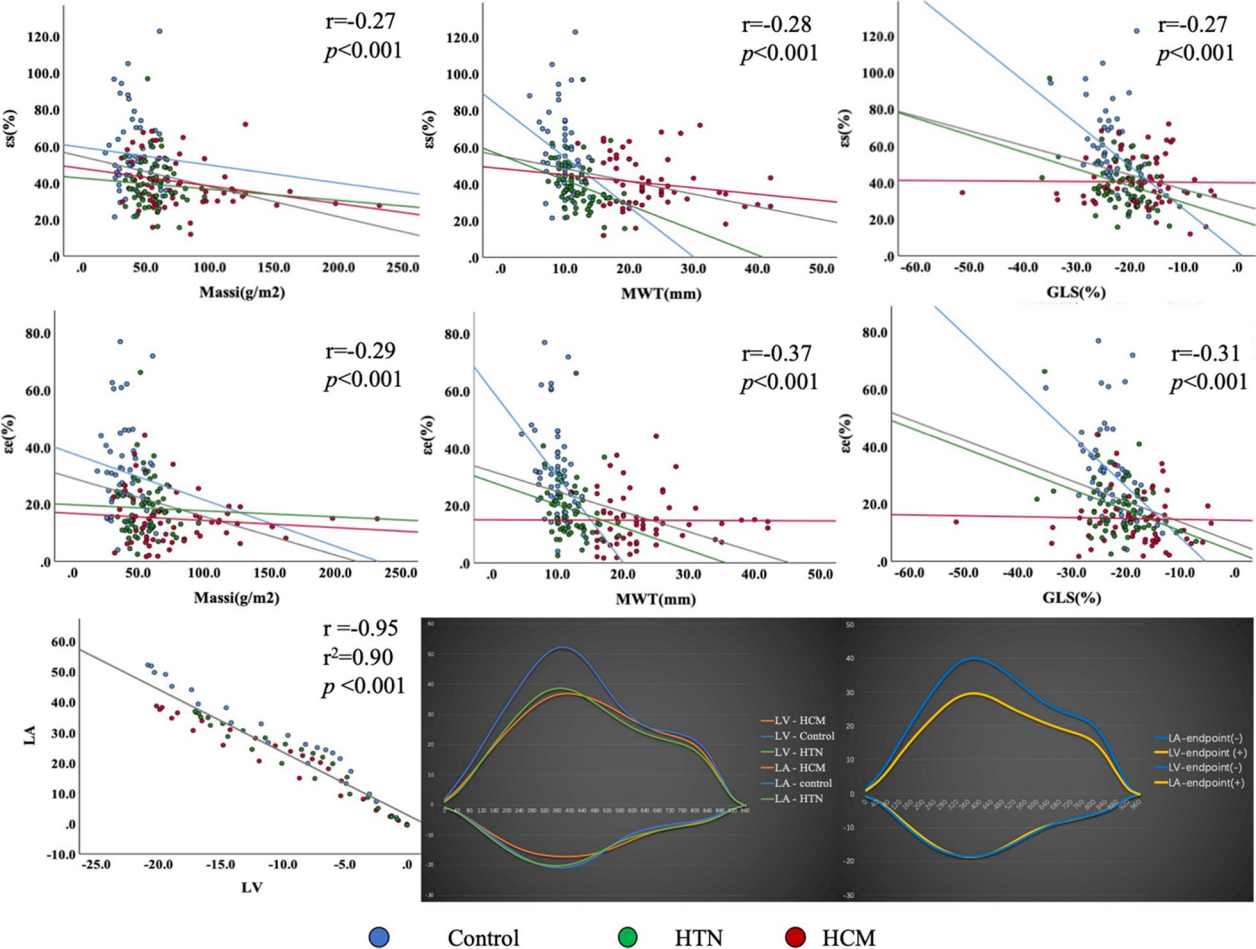
LA-LV coupling in all subjects. Pearson analysis was performed to investigate the correlations between left atrial strain with left ventricular strain during the whole cardiac cycle in all subjects. εs, total strain; εe, passive strain; GLS, global longitudinal strain; HTN, hypertension; HCM, hypertrophic cardiomyopathy; LA, left atrial; LV, left ventricular

LA volumes and dynamics as assessed by volumetric changes and deformation indexes were compared among the three groups in Table 3. The LA pre-contractile volumes and minimum LA volumes were the largest in HTN, followed by patients in HCM and controls (HTN vs. controls, LAV_pre-a_:44.4 ml ± 15.0 vs 34.0 ml ± 14.2, *p* < 0.01; LAV_min_: 23.4 ml ± 10.4 vs 17.5 ml ± 8.3, *p* = 0.01, respectively, Table 3). Left atrial reservoir and conduit functional parameters, including relative LAEF, strain and strain rate, showed significantly reduced in HTN and HCM cases as compared with those in controls (Figs. 2, 3). LA contractile function was preserved in HCM patients with normal LA size. LA active strain was significantly impaired in patients with HTN compared to the other groups (controls, 24.3% ± 8.9; HTN patients, 20.6% ± 6.1; HCM patients, 25.5% ± 9.7, *p* < 0.01) (Fig. 2). Although LA active EF also reflected decreased tendency in HTN patients, there was no statistical difference compared with the control.

**Table 3:** LA volumetric and deformation parameters assessed by CMR-FT

	HTN (n = 60)	HCM (n = 60)	Controls (n = 60)	*p* value^‡^
*LA volumetric parameters*				
V_max_, ml	57.8 ± 18.4	50.9 ± 17.8	49.1 ± 17.2	**0.02**
V_pre-a_, ml	44.4 ± 15.0*	39.9 ± 13.7	34.0 ± 14.2	**< 0.01**
V_min_, ml	23.4 ± 10.4*	21.2 ± 10.0	17.5 ± 8.3	**< 0.01**
*LA reservoir function*				
EF-total	59.9 ± 9.8*	58.6 ± 9.2*	65.1 ± 7.3	**< 0.01**
εs, %	39.2 ± 13.3*	40.3 ± 13.9*	54.1 ± 20.3	**< 0.01**
SRs, s-1	1.2 ± 0.4*	1.2 ± 0.4*	1.5 ± 0.4	**< 0.01**
*LA conduit function*				
EF-passive	22.8 ± 10.5*	20.8 ± 9.9*	31.1 ± 11.2	**< 0.01**
εe, %	18.6 ± 10.2*	14.8 ± 9.2*	29.9 ± 15.4	**< 0.01**
SRe, s-1	− 0.7 ± 0.3*	− 0.6 ± 0.3*	− 0.9 ± 0.4	**< 0.01**
*LA booster pump function*				
EF-active	48.1 ± 9.7	47.5 ± 11.0	49.1 ± 7.9	0.65
εa, %	20.6 ± 6.1*^†^	25.5 ± 9.7	24.2 ± 9.1	**< 0.01**
SRa, s-1	− 1.1 ± 0.4	− 1.2 ± 0.4	− 1.1 ± 0.4	0.22

Data are expressed as mean ± standard deviations. Bold values indicate statistical significance

HTN, hypertension; HCM, hypertrophic cardiomyopathy; LA, left atrial; EF, emptying fraction; εs, total strain; εe, passive strain; εa, active strain; SRs, total strain rate; SRe, passive strain rate; SRa, active strain rate

*Indicating p < 0.05 when compared with controls

^†^Indicating p < 0.05 when compared with HCM group

^‡^Significance of difference among three groups

### LA-LV coupling

There was a negative correlation between age and εs, εe, without εa in all participants. LA reservoir (εs) and conduit strain (εe) were both significantly associated with LV mass index (r = − 0.27, *p* < 0.001 and r = − 0.29, *p* < 0.001), maximal wall thickness (r = − 0.28, *p* < 0.001 and r = − 0.37, *p* < 0.001), GLS (r = − 0.27, *p* < 0.001 and r = − 0.31, *p* < 0.001), and GLSRd1 (r = 0.24, *p* < 0.01 and r = 0.28, *p* < 0.001) (Fig. 3). Further subgroup analysis indicated that LA reservoir and conduit strain were significantly associated with LV GLS in HTN group (all *p* < 0.01), and LA conduit strain was related to GLSRd2 in HCM group (*p* < 0.001, Table 4). Importantly, LA and LV time-strain curve has an extremely strong association in the entire cardiac cycle (r = − 0.95, *p* < 0.001) (Fig. 3).

**Table 4:** Pearson correlations of LA strain with clinical baseline, LV dysfunction and deformation in HTN and HCM patients without LA enlargement

	εs, %		εe, %		εa, %	
	r	*p* value	r	*p* value	r	*p* value
*HTN*						
Age, y	− 0.191	0.143	− 0.140	0.286	− 0.183	0.162
BSA, m^2^	− 0.231	0.075	− 0.171	0.191	− 0.218	0.094
SVI, ml/m^2^	0.285	**0.027**	0.315	**0.014**	0.094	0.474
CI, l/min/m^2^	0.306	**0.017**	0.322	**0.012**	0.129	0.327
Massi, g/m2	− 0.059	0.652	− 0.027	0.839	− 0.085	0.520
MWT, mm	− 0.272	**0.036**	− 0.206	0.115	− 0.248	0.056
GLS, %	− 0.363	**< 0.01**	− 0.370	**< 0.01**	− 0.173	0.187
GLSRs, s-1	− 0.284	**0.028**	− 0.271	**0.037**	− 0.167	0.203
GLSRd1, s-1	0.267	**0.039**	0.286	**0.027**	0.104	0.430
GLSRd2, s-1	0.154	0.239	0.164	0.210	0.062	0.640
*HCM*						
Age, y	− 0.123	0.351	− 0.294	**0.023**	0.102	0.437
BSA, m^2^	− 0.087	0.509	− 0.125	0.340	− 0.006	0.963
SVI, ml/m^2^	0.207	0.112	0.303	**0.018**	0.010	0.938
CI, l/min/m^2^	0.194	0.137	0.222	0.088	0.069	0.602
Massi, g/m2	− 0.274	**0.034**	− 0.107	0.417	− 0.292	**0.024**
MWT, mm	− 0.172	0.189	− 0.006	0.965	− 0.241	0.064
GLS, %	− 0.011	0.933	− 0.026	0.842	0.009	0.946
GLSRs, s-1	0.031	0.815	0.094	0.475	− 0.045	0.735
GLSRd1, s-1	0.109	0.407	0.173	0.186	− 0.007	0.956
GLSRd2, s-1	− 0.194	0.138	− 0.441	**< 0.001**	0.138	0.291

Bold values indicate statistical significance

LA, left atrial; LV, left ventricular; HTN, hypertension; HCM, hypertrophic cardiomyopathy; εs, total strain; εe, passive strain; εa, active strain; BSA, body surface area; SVI, Stroke volume index; CI, cardiac index; MWT, maximal wall thickness; GLS, global longitudinal strain; GLSRs, peak global longitudinal strain rate during LV contraction; GLSRd1, peak global longitudinal strain rate during early filling; GLSRd2, peak global longitudinal strain rate during atrial contractility at the end of LV diastole

### Outcomes

The mean follow-up duration was 6.8 years ± 2.1. There were 4 patients lost to follow-up. A total of 23 patients (19%) reached primary endpoints including 2 sudden cardiac deaths, 19 new-onset or worsening of HF to hospitalizations and 2 paroxysmal or persistent AF.

**Fig. 4:**
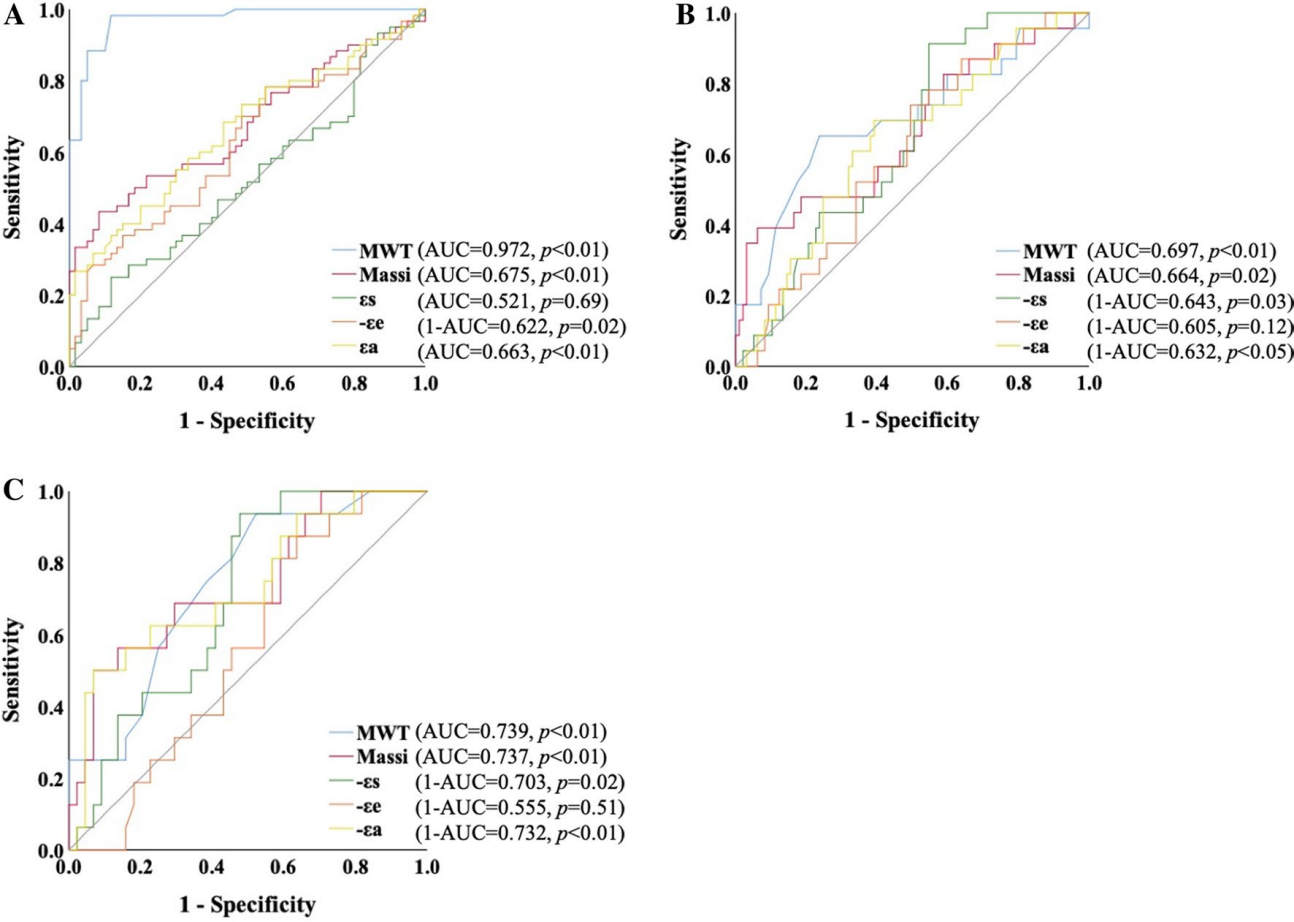
Receiver-operating characteristic curves for LV hypertrophy and LA strain were performed to estimate the area under the curves of these to differentiate HCM from HTN (**A**) and predict outcomes in HTN and HCM groups (**B**) and in HCM group (**C**). εs, total strain; εe, passive strain; εa, active strain; MWT, maximal wall thickness; LV, left ventricular; LA, left atrial; HTN, hypertension; HCM, hypertrophic cardiomyopathy

**Fig. 5:**
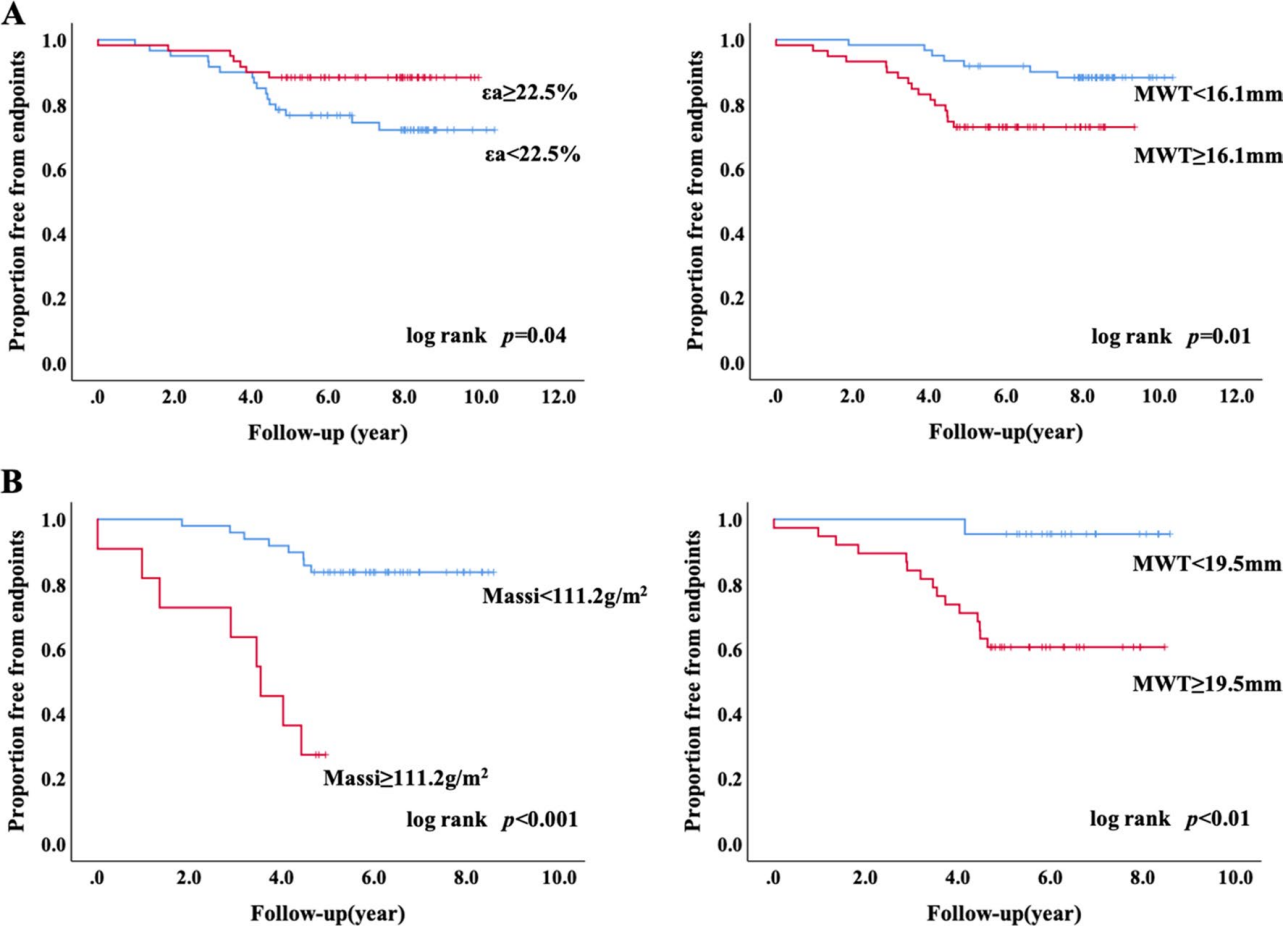
**A** Kaplan–Meier survival curves for primary endpoint development using LA ε_a_, and LVMWT in HTN and HCM patients. **B** Kaplan–Meier survival curves for primary endpoint development using left ventricular Massi and MWT in HCM patients. Composite event-free survival was significantly lower with εa < 22.5% (*p* = 0.04), LV MWT ≥ 16.1 mm (*p* = 0.01) in HTN and HCM patients, and with LV Massi ≥ 111.2 g/m^2^ (*p* < 0.001), MWT ≥ 19.5 mm (*p* < 0.01) in HCM patients. LA, left atrial; εa, active strain; LV, left ventricular; MWT, maximal wall thickness; HTN, hypertension; HCM, hypertrophic cardiomyopathy

LA strain was more sensitive than LV deformation for evaluate clinical adverse events (εs: 33.9% ± 7.5 vs. 41.2% ± 14.3, *p* = 0.02; εe: 13.6% ± 6.2 vs. 17.4% ± 10.4, *p* = 0.03; εa: 20.2% ± 6.0 vs. 23.7% ± 8.8, *p* = 0.07; GLS: − 19.4% ± 6.4 vs. − 20.0% ± 6.8, *p* = 0.70, respectively) (Fig. 3). Increased risks factors for primary endpoints were impaired LA reservoir strain, greater LV mass index and LV maximal wall thickness in all HCM and HTN patients (εs: HR: 0.960, 95% CI: 0.927–0.995, *p* = 0.025; Massi: HR: 1.024, 95% CI: 1.015–1.034, *p* < 0.001; MWT: HR: 1.111, 95% CI: 1.059–1.165, *p* < 0.001) (Table 5). In addition, patients with HCM experienced significantly higher rate of primary endpoint (log rank *p* = 0.014). Too few adverse events in the HTN group ceased to further assess clinical outcomes. Further univariate Cox analysis indicated LA contractile strain was solely associated with primary endpoints in the HCM group (HR: 0.924, 95% CI: 0.871–0.979, *p* = 0.007). ROC analysis indicated that LVMWT, Massi, εs and εa did not show diagnostic value for differentiation of HTN and HCM (Fig. 4A), but were capable to predict outcomes in all patients and in HCM group (Fig. 4B, C). Kaplan–Meier survival analysis revealed worse primary endpoint-free survival in patients with εa < 22.5% (*p* = 0.04), LVMWT ≥ 16.1 mm (*p* = 0.01) in all patients with HTN and HCM. For patients with HCM, LV Massi (≥ 111.2 g/m^2^, *p* < 0.001) and MWT (≥ 19.5 mm, *p* < 0.01) were also both related to the outcomes (Fig. 5).

**Table 5:** Results of univariate analyses in prediction of the clinical endpoint

	LR Chi2 (p value)	Wald	HR (95% CI)	*p* value
*Univariate Cox-HTN and HCM groups*				
Age, y	1.671 (0.196)	1.668	0.979 (0.949, 1.011)	0.196
Gender, male	0.089 (0.765)	0.089	0.877 (0.372–2.072)	0.765
BSA, m^2^	1.246 (0.264)	1.216	0.291 (0.032, 2.613)	0.270
LAV_-Max_, ml	0.044 (0.833)	0.044	0.998 (0.975, 1.020)	0.833
LAV_-pre-a_, ml	0.011 (0.918)	0.011	0.918 (0.973, 1.030)	0.918
LAV_-min_, ml	0.182 (0.670)	0.191	1.008 (0.971, 1.047)	0.662
LAEF-total	1.574 (0.210)	1.674	0.975 (0.938, 1.013)	0.196
LAEF-passive	0.289 (0.591)	0.290	0.989 (0.952, 1.028)	0.590
LAEF-active	1.404 (0.236)	1.487	0.978 (0.944, 1.014)	0.223
ε_s_, %	4.925 (0.026)	5.003	0.960 (0.927, 0.995)	**0.025**
ε_e_, %	2.783 (0.095)	2.859	0.956 (0.908, 1.007)	0.091
ε_a_, %	2.765 (0.096)	2.895	0.955 (0.905, 1.007)	0.089
Massi, g/m^2^	30.222 (< 0.001)	24.773	1.024 (1.015, 1.034)	**< 0.001**
MWT, mm	20.912 (< 0.001)	18.649	1.111 (1.059, 1.165)	**< 0.001**
GLS, %	0.116 (0.733)	0.116	1.011 (0.949, 1.077)	0.733
GLSRs, %	1.226 (0.268)	1.243	1.912 (0.612, 5.974)	0.265
GLSRd1, %	1.889 (0.169)	1.927	0.455 (0.150, 1.383)	0.165
GLSRd2, %	0.012 (0.912)	0.012	0.917 (0.197, 4.258)	0.912
*Univariate Cox-HCM group*				
εs, %	5.752 (0.016)	5.565	0.953 (0.915, 0.992)	**0.018**
εe, %	1.070 (0.301)	1.061	0.969 (0.913, 1.029)	0.303
εa, %	6.358 (0.012)	7.17	0.924 (0.871, 0.979)	**0.007**
Massi, g/m^2^	18.951 (< 0.001)	15.94	1.022 (1.011, 1.032)	**< 0.001**
MWT, mm	12.704 (< 0.001)	11.224	1.116 (1.047, 1.190)	**0.001**

Bold values indicate statistical significance

LA, left atrial; EF, emptying fraction; εs, total strain; εe, passive strain; εa, active strain; SRs, total strain rate; SRe, passive strain rate; SRa, active strain rate. LV, left ventricle; MWT, maximal wall thickness; GLS, global longitudinal strain; GLSRs, peak global longitudinal strain rate during LV contraction; GLSRd1, peak global longitudinal strain rate during early filling; GLSRd2, peak global longitudinal strain rate during atrial contractility at the end of LV diastole; HCM, hypertrophic cardiomyopathy

### Reproducibility

ICC of LA volumetric and deformation parameters for intra-observer variability ranged between 0.821 (0.631–0.978) (SRe) and 0.984 (0.743–0.998) (LV GLS), and for inter-observer variability ranged between 0.898 (0.709–0.964) (SRa) and 0.985 (0.969–0.993) (εe) (Table 6).

**Table 6:** Reproducibility of the LA and LV function analysis by CMR-FT

	Intra-observer		Inter-observer	
	ICC	95% CI	ICC	95% CI
εs, %	0.946	0.673–0.983	0.952	0.704–0.985
εe, %	0.950	0.690–0.984	0.985	0.969–0.993
εa, %	0.941	0.656–0.983	0.954	0.859–0.982
SRs, s-1	0.926	0.650–0.978	0.982	0.602–0.996
SRe, s-1	0.821	0.631–0.914	0.972	0.650–0.997
SRa, s-1	0.936	0.750–0.998	0.898	0.709–0.964
GLS, %	0.984	0.743–0.998	0.978	0.868–0.993
GLSRs, %	0.923	0.623–0.974	0.963	0.699–0.989
GLSRd1, %	0.958	0.794–0.985	0.962	0.823–0.987
GLSRd2, %	0.906	0.743–0.960	0.974	0.902–0.990

LA, left atrial; LV, left ventricle; ICC = intraclass correlation coefficient; CI, confidence interval; εs, total strain; εe, passive strain; εa, active strain; SRs, total strain rate; SRe, passive strain rate; SRa, active strain rate; GLS, global longitudinal strain; GLSRs, peak global longitudinal strain rate during LV contraction; GLSRd1, peak global longitudinal strain rate during early filling; GLSRd2, peak global longitudinal strain rate during atrial contractility at the end of LV diastole

## Discussion

In the current work, we explored LA function by CMR-FT in patients with HTN or HCM with normal LA size. The results provide several important insights: (1) Even without apparent LA enlargement, LA reservoir and conduit function were impaired in both HTN and HCM patients regardless of the different stage of LA booster pump dysfunction, which may suggest the different pathophysiological mechanisms of LA dysfunction in these two diseases. (2) LA strain is a promising biomarker to assess the hemodynamics dysfunction and impaired LA-LV coupling among different diseases. (3) At the early stage of cardiovascular disease, LA strain was a more sensitive and representative metric for evaluating myocardial impairments and more capable risk factor for prognosis, superior to LV strain.

Left atrial functional impairments have been previously reported in HTN and HCM using myocardial deformation imaging based on echocardiography and-more recently-using CMR-FT [26, 27]. Our data showed that LA reservoir and conduit function were impaired in HTN and HCM consistent with prior investigations concerning patients with LA enlargement [19, 26]. These abnormalities were significantly correlated with LVH and impaired LV GLS. The potential mechanisms contained increased LV wall stiffness, elevated LV filling pressure and impaired LA-LV coupling [6, 28]. We also noticed that HTN patients had lower LA active strain compared to HCM patients and the controls. In contrast to the HTN group, a trend of increased LA contractile function was present in patients with HCM, but this difference was not statistically significant. Preserved LA active function represents a compensatory mechanism to maintain stroke volume and LV filling with mild diastolic dysfunction and its deterioration reflects resultant reduction of LA compliance with LV fibrosis in a stage of “decompensation” [29, 30]. Therefore, LA dysfunctions at an earlier stage are predominantly provoked by the diastolic function abnormalities and may in turn provoke to LV fibrosis and systolic dysfunction [29]. Similarly, in this research, LA functions correlated more strictly with the severity of LV diastolic function (LV mass index, LV peak GLS during atrial contractility) in HCM. While in HTN, it correlates with the chronicity of disease (LV maximal wall thickness, LV deformation parameters reflecting systolic function). Although subjects performed preserved absolute values of LV mass index compared to the relative reference value about LVH in our study [31], we also found HCM patients had greater LV mass index and LV maximal wall thickness, reflecting LV diastolic function, compared to HTN patients. And HCM patients suffered from more serious longitudinal strain impairment, reflecting LV systolic function or fibrosis. In contrast, Dr. Lio and his colleagues described that HCM patients had more serious LA contractile dysfunction and larger LA volume than HTN patients without significant difference in LV mass index [10]. Therefore, it is important to evaluate pathophysiological mechanisms and LA-LV coupling in patients with HTN or HCM. Previous studies demonstrated that LA dysfunction could be initiated by pressure-related LV diastolic dysfunction imposed by chronic hypertension before the clinically apparent LVH in HTN [26, 32]. While HCM is the most common inheritable heart disorder [33], characterized by myocyte hypertrophy, disarray, and fibrosis [34, 35]. The pathological mechanism of HCM reported by studies in animal models has found significant and upregulation of genes involved in extracellular matrix synthesis [36]. These genetic pathways were activated to make increase in extracellular matrix, and then to give rise to LVH or myocardial fibrosis developed [37]. In this context, it is interesting to speculate that LA function derived from CMR-FT may be a promising ongoing biomarker to assess the hemodynamics dysfunction and impaired LA-LV coupling among diseases with pathologic LVH in the early stage.

Although, previous studies roughly demonstrated impaired LA function without LA enlargement, assessed by global longitudinal LA strain and LA volumetric indices, few studies have been completely focused on the association between LA function and prognostic implications in the scenario of various etiology [4, 8, 9, 26]. In our cohort, although no differences were noted in LA volumetric parameters, impaired LA reservoir and contractile strain are significantly worse in patients admitted in primary endpoints, within the normal range of parameters representing LV hypertension. This finding extends finding of prior prospective research with 257 post-MI patients who suffered different grades of diastolic dysfunction [38]. It supported the concept that LA strain, derived of both echo- and CMR, has diagnostic utility for stratifying presence and severity of diastolic dysfunction. In addition, we found patients with abnormal LA contractile strain (< 22.5%) experienced significantly higher rate of adverse clinical events. Investigation in HF subjects proposed that LA declined contractility, coinciding with adverse changes in reduced intrinsic contractility, remodeling, apoptosis, collagen matrix turnover and myosin isoform expression, may contribute to greater burden of AF in HF patients with preserved LVEF [39]. It has been suggested that LA strain could potentially identify ambulatory patients with cardiovascular events at a higher risk of overt HF performances [40]. Interestingly, there was no significantly prognostic value in LV strain in this study. In recently, there are large of studies regarding the prognostic efficacy of LA strain and LV GLS with contradictory results. Some researchers reported that LA reservoir strain and conduit strain were independent predictors of major adverse cardiac events following ST-segment elevation myocardial infarction after adjustment for established clinical and CMR markers of cardiovascular risk including GLS [41, 42]. Negishi et al. demonstrated that LA booster pump strain was an independent and incremental predicted marker of arrhythmias over GLS in 124 patients with non-ischemic dilated cardiomyopathy [43]. In contrast, in a multicenter prospective study that included 1110 patients with myocardial infarction referred for invasive coronary angiography, they found that LA reservoir strain did not add further information in regard to adverse outcome, when readily obtained GLS and maximum LA volume were known [44]. Our results suggest that underlying mechanism of LA-LV coupling is promisingly urgent issue and larger studies are necessary to explore the prognostic role of myocardial phasic function during the different stages of cardiovascular diseases. Although, further studies are needed to support our findings and validate the availability of this tool, the application of cine CMR-FT for assessment and risk stratification in these populations undergoing clinical routine examination, particularly in the early stage, may become a short-term reality [45].

There are some limitations in the present study. Firstly, in consideration of the major aim, limited cases in HCM with normal LA size who came to see the doctors, the sample size in our study was relatively small. the correlations of LA-LV measurements are weak-to-fair. Despite age-related correlation and survival analyses, difference in age between groups may confound the conclusions. More and larger scale studies are needed to confirm these findings. Secondly, due to the relatively early disease stage with a few cardiovascular events in this cohort, we acknowledge that only univariable Cox regression analyses were performed at a single time point. Thus, the power of prognostic analysis is limited, and we cannot confirm whether LA strain is an independent risk factor for primary endpoint. Thirdly, differences of strain measurements caused by various CMR-FT vendors cannot be excluded. So standardized postprocessing methods would be desirable to facilitate comparative analysis of LA deformation and may reduce inter-vendor variability.

## Conclusions

CMR-FT-derived strain is a promising and robust tool in demonstrating impaired LA mechanics, quantifying LA dynamics and underlining the importance of LA-LV coupling in patients with HTN and HCM, whose left atrium is even in the normal size. The corresponding LA dysfunctions were strictly associated with clinical implication and provided prognostic utility at the early stage of HTN and HCM superior to GLS. Further multicenter, large scale and prospective studies are need to confirm and verify our findings.

## Data Availability

The datasets used and/or analyzed during the current study are available from the corresponding author on reasonable request.

## Abbreviations

LA: Left atrial
HTN: Hypertension
HCM: Hypertrophic cardiomyopathy
AF: Atrial fibrillation
HF: Heart failure
LVH: Left ventricular hypertrophy
CMR: Cardiovascular magnetic resonance
STE: Echocardiographic speckle tracking
CMR-FT: CMR feature tracking
SSFP: Steady-state free precession
LAEF: Left atrial emptying fraction
SR: Strain rate
εs: Total strain
εa: Active strain
εe: Passive strain
GLS: Global longitudinal strain
ICC: Intraclass correlation coefficient
ANOVA: One-way analysis of variance
HRs: Hazard ratios
ROC: Receiver operating characteristic
AUC: Area under the curves
MWT: Maximal wall thickness
